# Effects of pentoxifylline on mouse oocytes maturation and quality *in vitro*

**DOI:** 10.22038/ijbms.2024.77926.16856

**Published:** 2025

**Authors:** Junjiao Wu, Jianbo Wu, Yanyan Xu

**Affiliations:** 1 Department of Obstetrics-gynecology, The First Affiliated Hospital of Fujian Medical University, Fuzhou, 350005, China

**Keywords:** Chromosomes, Cortical granules, Oocyte maturation, Pentoxifylline, Reactive oxygen species, Spindle apparatus

## Abstract

**Objective(s):**

To investigate the impact of Pentoxifylline (PTX) on the *in vitro* maturation (IVM) of mouse oocytes and its effect on oocyte quality.

**Materials and Methods:**

This experimental study involved culturing mouse oocytes in an IVM medium with varying PTX concentrations (0-100 μM). Post-culture, oocytes were assessed for nuclear and cytoplasmic maturation and quality indicators, including germinal vesicle breakdown (GVBD), first polar body extrusion (PB1E), cortical granules (CGs) distribution, spindle structure, chromosome alignment, and intracellular reactive oxygen species (ROS) levels.

**Results:**

Treatment with PTX at 10, 25, and 50 μM concentrations significantly enhanced the nuclear maturation rates of oocytes. The optimal concentration was found to be 10 μM, as it resulted in the most favorable cytoplasmic maturation, characterized by improved distribution of CGs, spindle structure, and chromosome alignment. Additionally, treatment with 10 μM PTX effectively reduced reactive oxygen species (ROS) levels.

**Conclusion:**

PTX supplementation at specific concentrations enhances mouse oocyte maturation and quality, potentially by facilitating CG distribution, spindle integrity, and chromosome alignment and by reducing ROS production.

## Introduction

*In vitro* maturation (IVM) of immature oocytes is a growing topic in Assisted Reproductive Technology (ART), and it has become increasingly popular in treating human infertility in recent years. Experiments have demonstrated, however, that mature oocytes *in vitro* have a much lower developing ability than mature oocytes *in vivo* (1-3). *In vivo*, the inhibition of nuclear maturation by follicular fluid results in an optimal balance of nuclear and cytoplasmic maturation (4, 5). *In vitro*, the oocyte was removed from the follicular inhibition environment and placed in a culture medium to resume meiosis prematurely without sufficient cytoplasmic maturation (6, 7). As a result of the disorganized nuclear and cytoplasmic maturation, developmental competence is reduced, resulting in a low rate of blastocyst development (8). Therefore, numerous studies expect to increase the cytoplasmic maturation of oocytes by delaying meiosis recovery during IVM (9-11). 

Pentoxifylline (PTX) is an old medicine extensively used for treating vascular diseases (12). PTX belongs to the family of non-specific phosphodiesterase (PDE) inhibitors that lead to an increase in cyclic adenosine monophosphate (cAMP) levels, which are known to enhance sperm motility (13, 14). Moreover, it is an anti-oxidant that reduces oxidative stress-induced sperm damage (15, 16). 

cAMP has been shown to play a pivotal role in the progression of meiosis in various studies (17-19). PDE inhibitors elevate cAMP levels, leading to maintenance of meiotic arrest at the diplotene stage and oocyte maturation, ovulation, fertilization, and pregnancy rates (11, 19-21). The reversible inhibitory effects of PDE inhibitors on oocyte maturation and ovulation without altering the menstrual cycle make them promising candidates for non-hormonal therapeutic contraceptive drugs with minimal adverse effects (11). PTX is a member of the PDE inhibitors, although few studies have explored its impact on the *in vitro* maturation of mouse oocytes. 

Anti-oxidants regulate ROS, which are necessary for cellular function. Follicles, oocytes, endometrium, and their surroundings are all affected by ROS (22). Overproduction of ROS causes oxidative stress and apoptosis, reducing oocyte quality and developmental potential (23). Therefore, ROS levels are closely linked to oocyte quality. A balance between anti-oxidants and ROS regulates oocyte maturation and quality (24). PTX is an anti-oxidant that reduces the maturation of rat-denuded oocytes cultivated *in vitro*, perhaps by lowering the quantity of ROS (25).

In light of the above, the objective of this present study was to fill a research problem about the impact of PTX on the *in vitro* maturation process of mouse oocytes. Here, we explored the effects of PTX supplementation on the rates of GVBD and PB1E of mouse oocytes during IVM. Additionally, we examined the influence of PTX on the distribution of CGs, spindle structure, chromosome alignment, and intracellular levels of ROS. These findings may elucidate potential mechanisms underlying the beneficial effects of PTX on the maturation and quality of mouse oocytes.

## Materials and Methods

### Mouse and reagents

All animal experiments were performed in strict accordance with the SPF Animal feeding measures of Fujian Medical University’s Experimental Animal Research Center and the Ethics Committee (Permit Number: SCXK 2017-0012). Unless otherwise noted, all reagents were purchased from Sigma-Aldrich (USA). ICR mice were housed in a temperature-controlled environment on a 12-hr light/12-hr dark cycle, fed a standard diet, and fed adaptively for one week.

### Oocyte collection and IVM

Female ICR mice aged 5-6 weeks were injected intraperitoneally with 10 IU PMSG and sacrificed 46–48 hr later by cervical dislocation for oocyte collection. Oocytes were collected from ovarian follicles in an immature stage (germinal vesicle) in M2 medium, then treated with hyaluronidase and denuded of cumulus cells using a fine bore glass pipette. Only denuded oocytes with full shape and complete germinal vesicles were used for the following experiments.

M16 medium was used as a maturation medium. Ten to twenty oocytes were cultured in 50 μL drops of maturation medium.

### PTX supplementation

PTX was dissolved in the medium at the final concentration of 2.5 μM, 5 μM, 10 μM, 25 μM, 50 μM, and 100 μM and sterilized by filtration.

### Assessment of oocyte maturation by first polar body extrusion

Oocyte maturity was assessed 24 hr after IVM culture, and the oocytes with a first polar body were considered mature under a stereoscopic microscope. The stages of nuclear maturation were GV (germinal vesicle stage), GVBD (when the GV was not visible), and MII (first polar body observed in the perivitelline space). After three replications, the data was analyzed.

### Cortical granule staining

All steps are carried out at room temperature unless otherwise specified.

MII oocytes were washed in DPBS+0.3% PVP and fixed in 4 percent paraformaldehyde for 20 min. The fixed oocytes were removed, exposed to tabletop acid for 20 sec, and washed with CGs blocking solution (DPBS+0.3%BSA+l00mM Glycine). The washed oocytes were permeabilized with 1% TritonX-100 dissolved in DPBS for 30 min and then stained for 30 min with FITC-Lens culinary agglutinin (FITC-LCA). Subsequently, oocytes were washed with a CG-blocking solution. Then, DNA was stained by 4, 6-diamidino-2-phenylindole (DAPI) for 10 min. After that, the stained oocytes were mounted on microscope slides visualized under a fluorescence microscope and analyzed by Image-Pro Plus. 

### Spindle structure and chromosome staining

MII oocytes were washed in DPBS+0.3% PVP and fixed with 4% paraformaldehyde for 20 min. The washed oocytes were permeabilized in DPBS for 30 min with 1 percent TritonX-100 dissolved. Oocyte spindles were stained by sequentially incubating the oocytes on monoclonal mouse anti-alpha-tubulin and FITC-goat anti-mouse antibodies. Chromosomes were stained with DAPI and mounted on microscope slides after being thoroughly washed; Image-Pro Plus was used to examine the results.

### Intracellular ROS level

Following 24 hr of IVM, MII oocytes were collected from the control and treatment groups (10 μM), washed in DPBS, and incubated in 10µM of DCFH-DA in DPBS for 15 min in the dark. After incubation, the MII oocytes were washed completely in DPBS, placed in a well of a hanging drop slide in 20 µl of DPBS, and observed under a fluorescence microscope. Image-Pro Plus was used to evaluate the images of the stained MII oocytes, which were obtained with the same conditions for all groups.

### Statistical analysis

All of the experiments were repeated at least three times. The data in this study were performed using SPSS and GraphPad software. Oocyte maturation in different concentrations was analyzed using one-way ANOVA followed by LSD-t test. Differences in groups of CG distribution, spindle structure, and chromosome alignment were performed with the chi-square. Comparisons of intracellular ROS levels were performed with a t-test. The images presented were analyzed using Image-Pro Plus software. All data was reported as mean values ± standard error (SEM), with *P*-values less than 0.05 considered statistically significant.

## Results

### Meiotic progression of mouse oocytes during IVM

**Figure 1:**
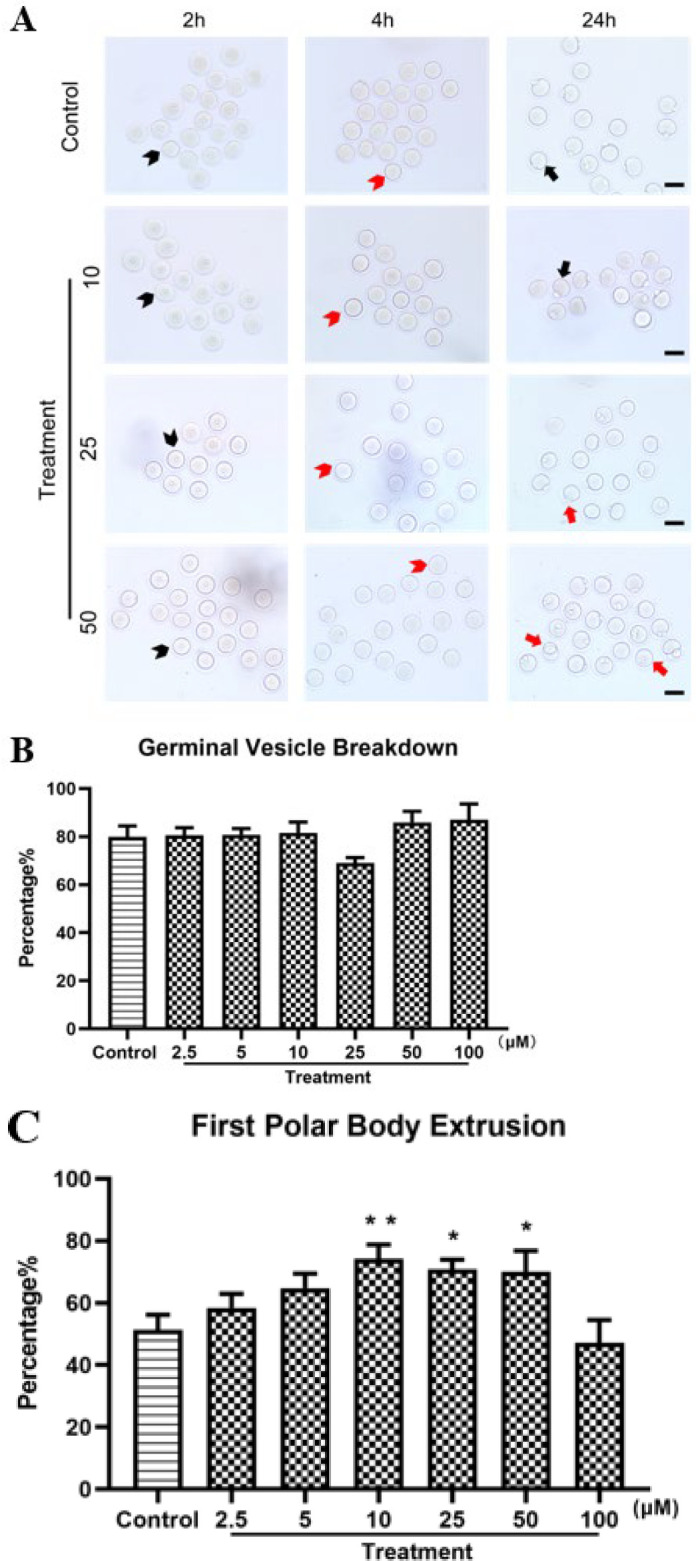
Effect of PTX on mouse oocytes IVM

We assessed the impact of varying PTX concentrations (0, 2.5, 5, 10, 25, 50, and 100 μM) on the meiotic progression of mouse oocytes during IVM. After a 4-hr culture period, the GVBD rate showed a non-significant decrease in the 25 μM PTX group compared to other groups (*P*=0.084; Figure 1A, B). Following a 24-hr culture, the PB1E rate was significantly higher in the 10, 25, and 50 μM PTX groups than in the control group (*P*<0.01; *P*<0.05; *P*<0.05; Figure 1A, C). In contrast, the 100 μM group exhibited fewer oocytes reaching the MII stage.

### Cortical granules distribution

**Figure 2:**
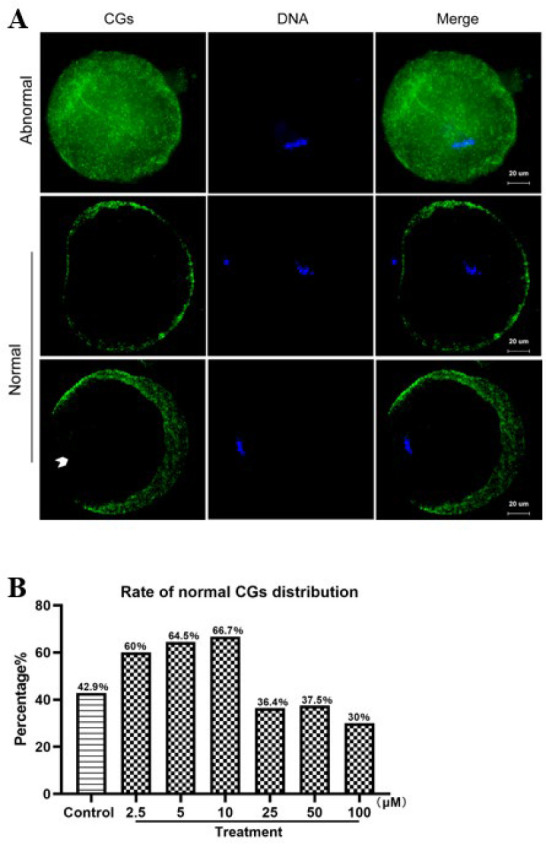
Effect of PTX on CGs distribution of mouse oocytes IVM

After 24 hr, CG distribution was evaluated under a fluorescence microscope. The groups treated with 2.5, 5, and 10 μM PTX displayed higher rates of normal CG distribution, with most CGs migrating to the cortex or forming a cortical granules-free domain (CGFD) (Figure 2B). In contrast, CGs in other groups remained distributed throughout the oocyte cytoplasm (Figure 2A).

### Spindle structure and chromosome alignment

**Figure 3:**
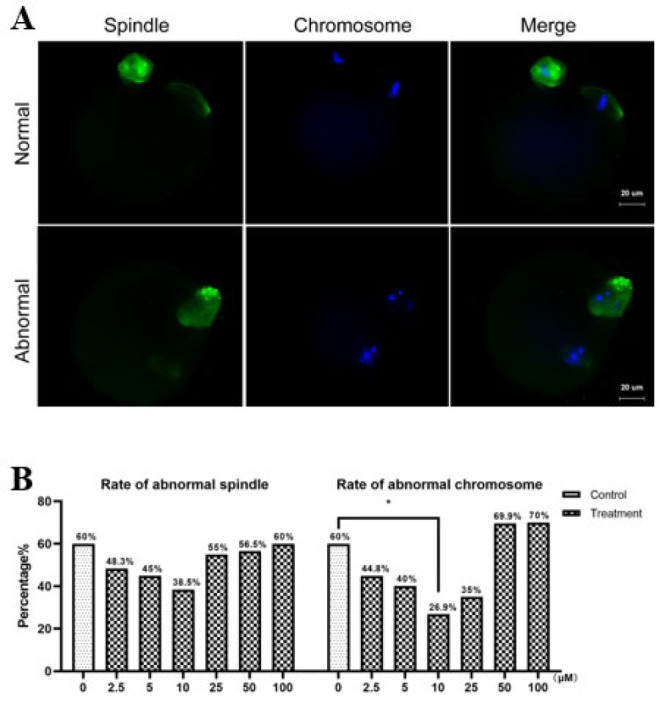
Effect of PTX on mouse oocytes spindle structure and chromosome alignment IVM

At the conclusion of IVM, spindle structure and chromosome alignment were analyzed (Figure 3A). A reduction in oocytes with dispersed spindles and misaligned chromosomes was observed in the 2.5, 5, 10, and 25 μM PTX groups. Notably, the 10 μM PTX group showed well-aligned chromosomes and fewer aberrant spindles compared to other groups, suggesting an optimal concentration for enhancing cytoplasmic maturation (*P*=0.154, *P*<0.05; Figure 3B).

### Intracellular ROS level

**Figure 4:**
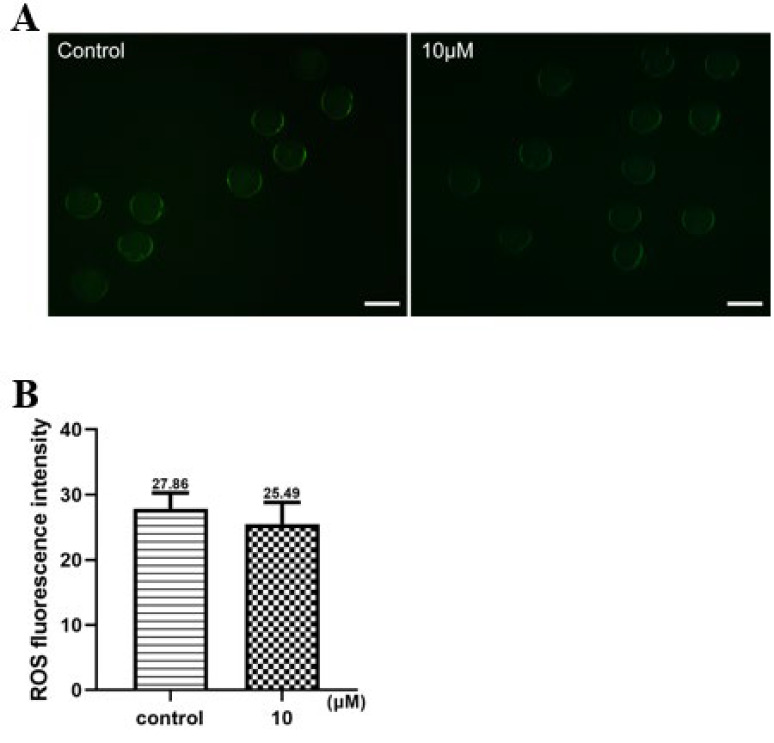
Effect of PTX on mouse oocytes intracellular ROS IVM

The effect of PTX on oxidative stress was determined by measuring intracellular ROS levels post-IVM under a fluorescent microscope. A decrease in ROS signals was noted in oocytes treated with 10 μM PTX, although this did not reach statistical significance (*P*=0.231; Figure 4 A, B).

## Discussion

PTX is commonly utilized in male ART to assist in the selection of viable sperm and enhance sperm motility prior to intracytoplasmic sperm injection (ICSI) in patients with asthenozoospermia (14, 16, 26). However, little was known about the effect of PTX on mouse oocytes *in vitro* maturation. In this study, our primary objective was to investigate whether PTX affects the nuclear maturation of mouse oocytes. Additionally, we explored the influence of PTX on the cytoplasmic maturation of mouse oocytes as part of our secondary investigation. Finally, a series of experiments were conducted to elucidate the underlying mechanism by which PTX promotes *in vitro* maturation of mouse oocytes.

Oocyte maturation is divided into two stages: nuclear and cytoplasmic maturation (27). The stages of nuclear maturation were GV (germinal vesicle stage), GVBD (when the GV was not visible), and MII (first polar body observed in the perivitelline space). We examined the GVBD and PB1E rates in mouse oocytes to focus on whether PTX influenced nuclear maturation (28). According to our findings, the GVBD rate fell only in the 25 μM group, while the PB1E rate reduced only in the 100 μM group. Additionally, as the concentration of PTX increased in low-concentration groups (2.^5^, ^5^, ^10^), more oocytes of the first polar body were extruded. This contradicts previous studies that specific PDE inhibitors reversibly hinder spontaneous departure from diplotene arrest and oocyte development by raising cyclic nucleotide levels (11). According to Chaube’s findings, PTX greatly reduced the maturation of denuded rat oocytes, and 1 mM of PTX significantly decreased the rate of maturation (*P*<0.001) (29). Gupta and Chaube, on the other hand, found that rolipram (PDE 4D inhibitors) did not prevent spontaneous meiotic resumption in denuded oocytes of rats cultivated *in vitro*(10), which was analogous to our findings. These perplexing results might be explained by the fact that PTX has a similar affinity for PDE4 (11, 30) even though PDE4s are plentiful in somatic granulosa cells but not in the oocyte (20, 31). As a result, PDE inhibitors can either inhibit or accelerate oocyte maturation, depending on experimental settings and the species. Consequently, our findings imply that PTX improves nuclear maturation in mouse oocytes at suitable concentrations. 

Cytoplasmic maturation is also necessary to allow normal development of embryos (19, 32). The distribution of CGs and the cytoskeleton dynamics are crucial in cytoplasmic maturation and oocyte quality (33, 34). CGs are oocyte cytoplasmic organelles that secrete substances that prevent polyspermy during acrosome and cortical reactions (35, 36). CGs are limited to the innermost part of the cytoplasm during the GV stage, then migrate to the cortex or form a cortical granules-free domain (CGFD) throughout maturation (37, 38). Moreover, CG migration depends on cytoskeleton function, and orderly meiosis in oocytes necessitates precise control of spindle structure and chromosomal alignment (32, 39). Any mistake in this process can lead to aneuploid eggs, which are the main cause of pregnancy loss and developmental disabilities in humans (40). As a result, mature oocytes should have spindles and chromosomes that are morphologically normal. Here, we discovered that supplementing with PTX during IVM reduced the incidence of aberrant spindle and chromosomal formation while also increasing the rate of normal CG distribution. The findings suggest that PTX supplementation enhances cytoplasmic maturation and quality during *in vitro* oocyte maturation, partly by facilitating normal distribution of CGs, maintaining spindle structure, and promoting chromosome alignment. Based on the findings, the best PTX concentration for mouse oocytes IVM is 10 μM. Consequently, the treatment concentration for the following research was 10 μM PTX.

Oocyte maturation and quality are regulated by a balance between anti-oxidants and reactive oxygen species (ROS) (24). The *in vitro* cell culture environment produces a considerable amount of ROS due to the high oxygen concentration than in the *in vivo* environment (41). The physiological concentration of ROS has been demonstrated to play a vital role in normal cell activity, while high concentrations of ROS can be detrimental to cells (42). Excessive ROS production can lead to oxidative stress, disrupting the meiosis process (43). In our study, the rate of aberrant spindles with misaligned chromosomes and abnormal CG distribution was more significant in the control oocytes with elevated ROS levels than in the PTX oocytes supplemented with 10 μM. These findings support Premkumar and Chaube’s previous research, indicating that PTX effectively mitigates excessive ROS production during *in vitro* oocyte maturation (25).

Throughout our research, at low concentrations (2.5 to 10 μM), PTX showed a dose-dependent positive effect, promoting the maturation of oocytes. In contrast, at higher concentrations (25 to 100 μM), the effect of PTX showed a dose-dependent negative effect, inhibiting oocyte maturation. Within the concentration range of 2.5 to 10 μM, as the concentration of PTX increases, the rates of GVBD and PB1E in oocytes significantly improve, indicating that PTX promotes nuclear maturation of oocytes within this concentration range. However, within the concentration range of 25 to 100 μM, the effect of PTX is the opposite. In the 100 μM PTX group, there was a decrease in oocytes progressing to the MII stage; concurrently, an increase in the concentration of PTX was correlated with a greater number of abnormal first polar bodies (Figure 1A). This biphasic response may be related to the inhibitory effect of PTX on PDE. PTX promotes oocyte maturation at low concentrations by inhibiting PDE and increasing cAMP levels. At high concentrations, PTX may over-suppress PDE, leading to excessively high levels of cAMP, which interferes with the normal maturation process of oocytes (44, 45). Additionally, high concentrations of PTX may be toxic to cells, affecting cellular structure and function, thereby hindering oocyte maturation. Further research is needed to understand the mechanisms underlying the observed biphasic response fully and determine the optimal use of PTX in the context of oocyte maturation and fertility treatments.

(A) After culture for 2, 4, and 24 hr *in vitro*, pictures demonstrated distinct morphologies of mouse oocytes without PTX (control) or with different concentrations of PTX (treatment). Representative oocytes at various stages of development are shown here. The black arrowhead indicates GV, the red arrowhead indicates GVBD, the black arrow indicates MII, and the red arrow indicates matured oocytes with an abnormal first polar body. Scale bar = 100 μm. (B, C) After culture for 4 and 24 hr in vitro, bar graphs revealed the percentage of GVBD and PB1E of mouse oocytes. The data is expressed as means ± SEM. * *P*<0.05, * * *P*<0.01 versus the control.

PTX: Pentoxifylline; IVM: *In vitro* maturation; GV: Germinal vesicle; GVBD: Germinal vesicle breakdown

(A) Representative CGs stained with Lens culinaris agglutinin (LCA) (green) and chromosomes stained with 4, 6-diamidino-2-phenylindole (DAPI) (blue) were shown in the images. The white arrowhead indicates CGFD. Scale bar = 20 μm. (B) Percentage of oocytes having a normal CGs distribution was shown in bar graphs.

PTX: Pentoxifylline; CGs: Cortical granules; IVM: *In vitro* maturation

(A) Images showed a representative spindle stained with anti-α-tubulin antibody (green) and chromosomes stained with DAPI (blue). Scale bar = 20 μm. (B) Percentage of oocytes with an abnormal spindle and chromosome was shown in bar graphs. * *P*<0.05 versus the control.

PTX: Pentoxifylline; IVM: *In vitro* maturation; DAPI: 4, 6-Diamidino-2-phenylindole

(A) The pictures presented representative oocytes of ROS stained with DCFH-DA (green). Scale bar = 100 μm. (B) Fluorescence intensity of ROS per each oocyte was represented by bar graphs.

PTX: Pentoxifylline; ROS: Reactive oxygen species; IVM: *In vitro* maturation

## Conclusion

In summary, supplementing mouse oocytes with 10 μM PTX significantly improved their maturation and quality *in vitro*. PTX’s beneficial effects may be mediated by enhancing CG distribution, spindle structure, and chromosome alignment, which reduces excessive ROS production.

## Declaration

We have not used any AI tools or technologies to prepare this manuscript.
